# The Asian Cohort for Alzheimer's Disease (ACAD) Study: 2025 Update

**DOI:** 10.1002/alz70860_099372

**Published:** 2025-12-23

**Authors:** Pei‐Chuan Ho, Boon Lead Tee, Clara Li, Yian Gu, Jennifer S. Yokoyama, Dolly Reyes‐Dumeyer, Zoe D. McManus, Kelley M. Faber, Wan‐Ping Lee, Yeunjoo E. Song, Marian Tzuang, Badri N. Vardarajan, Hyun‐Sik Yang, Yun‐Beom Choi, Howard H. Feldman, Josh D Grill, Victor W Henderson, Ging‐Yuek Robin Hsiung, Richard Mayeux, Howard J. Rosen, Rohit Varma, Tatiana M. Foroud, Guerry M. Peavy, Walter W. Kukull, Haeok Lee, Wai Haung Yu, Helena C Chui, Gyungah R Jun, Van Ta Park, Tiffany W Chow, Li‐San Wang, The Asian Cohort for Alzheimer's Disease Study

**Affiliations:** ^1^ University of Pennsylvania Perelman School of Medicine, Philadelphia, PA, USA; ^2^ Memory and Aging Center, UCSF Weill Institute for Neurosciences, University of California, San Francisco, San Francisco, CA, USA; ^3^ Icahn School of Medicine at Mount Sinai, New York, NY, USA; ^4^ Cognitive Neuroscience Division, Columbia University, New York, NY, USA; ^5^ University of California, San Francisco, San Francisco, CA, USA; ^6^ Department of Neurology, College of Physicians and Surgeons, Columbia University, New York, NY, USA; ^7^ National Centralized Repository for Alzheimer's Disease and Related Dementias, Indianapolis, IN, USA; ^8^ Department of Population and Quantitative Health Sciences, Case Western Reserve University School of Medicine, Cleveland, OH, USA; ^9^ University of California San Francisco School of Nursing, San Francisco, CA, USA; ^10^ Department of Neurology, Columbia University, New York, NY, USA; ^11^ Brigham and Women's Hospital, Harvard Medical School, Boston, MA, USA; ^12^ Englewood Health, Englewood, NJ, USA; ^13^ Alzheimer's Disease Cooperative Study, Department of Neurosciences, University of California San Diego, La Jolla, CA, USA; ^14^ University of California, Irvine, Irvine, CA, USA; ^15^ Stanford University, Palo Alto, CA, USA; ^16^ University of British Columbia, Vancouver, BC, Canada; ^17^ Department of Neurology, Vagelos College of Physicians and Surgeons, Columbia University, New York, NY, USA; ^18^ Department of Neurology, Memory and Aging Center, University of California San Francisco, San Francisco, CA, USA; ^19^ Southern California Eye Institute, CHA Hollywood Presbyterian Medical Center, Los Angeles, CA, USA; ^20^ Department of Neurology, Indiana University School of Medicine, Indianapolis, IN, USA; ^21^ University of California San Diego, Department of Neurosciences, La Jolla, CA, USA; ^22^ Department of Medicine, University of Washington, Seattle, WA, USA; ^23^ Meyers College of Nursing, New York University, New York, NY, USA; ^24^ University of Toronto, Toronto, ON, Canada; ^25^ Department of Neurology, Keck School of Medicine, University of Southern California, Los Angeles, CA, USA; ^26^ Biomedical Genetics Section, Department of Medicine, Boston University Chobanian & Avedisian School of Medicine, Boston, MA, USA

## Abstract

**Background:**

Asian Americans and Asian Canadians (ASACs) are the fastest‐growing minority group in the US and Canada, yet they are under‐representative in Alzheimer's disease (AD) research. To address the need of culturally appropriate clinical protocols and community‐based recruitment approaches for ASACs, the Asian Cohort for Alzheimer's Disease (ACAD), the first large dementia genetics cohort focusing on Chinese, Korean, and Vietnamese, launched in 2021 to investigate genetic and non‐genetic risk factors for AD among ASACs. Our clinical and community‐based participatory research (CPBR) scientists have a long collaborative history and diverse cultural and scientific training backgrounds: both are critical in leading AD and CBPR research.

**Method:**

Upon receipt of an NIA U19 grant in 2023, ACAD has expanded to 9 recruiting sites, a coordinating site, and an analysis site with a centralized data management system. Gathering feedback from its pilot phase, ACAD updated the study protocol including community outreach and recruitment strategies, the data collection packet, pre‐screening and sample collection procedures, and in English, Chinese (Mandarin and Cantonese), Korean, and Vietnamese. The updated DCP includes more neuropsychological tests and cultural tailored lifestyle questionnaires with an emphasis on immigration experiences. To ensure consistency, ACAD implemented a training curriculum for data/sample collect and for culturally appropriate recruitment approaches in collaboration with community partners, clinics, and nursing homes serving Asian communities.

**Result:**

As of January 2025, ACAD has consented 1,173 participants and has additional 1,920 people on the interest list. The follow‐up visits are expected to begin in Spring 2025, providing insights into conversion to dementia and disease progression. ACAD has developed a cultural appropriate data collection protocol, which is robust for both in‐person and virtual assessments. A comprehensive training curriculum covering outreach, prescreening, consenting, data and sample collects, and data entry is in place to ensure study rigor.

**Conclusion:**

The ACAD team (including community partners) have demonstrated the feasibility of recruiting ASACs in clinical research. With an expansion plan and in collaboration with other AD research focuses on racial minority populations, insights from ACAD may identify potential novel, population‐specific therapeutic pathways for AD.

